# Nanoparticle chemisorption printing technique for conductive silver patterning with submicron resolution

**DOI:** 10.1038/ncomms11402

**Published:** 2016-04-19

**Authors:** Toshikazu Yamada, Katsuo Fukuhara, Ken Matsuoka, Hiromi Minemawari, Jun'ya Tsutsumi, Nobuko Fukuda, Keisuke Aoshima, Shunto Arai, Yuichi Makita, Hitoshi Kubo, Takao Enomoto, Takanari Togashi, Masato Kurihara, Tatsuo Hasegawa

**Affiliations:** 1 National Institute of Advanced Industrial Science and Technology (AIST), AIST Tsukuba Central 5, Tsukuba 305-8565, Japan; 2 Department of Applied Physics, The University of Tokyo, Tokyo 113-8656, Japan; 3 Tanaka Kikinzoku Kogyo K. K., Tsukuba 300-4247, Japan; 4 Department of Material and Biological Chemistry, Yamagata University, Yamagata 990-8560, Japan

## Abstract

Silver nanocolloid, a dense suspension of ligand-encapsulated silver nanoparticles, is an important material for printing-based device production technologies. However, printed conductive patterns of sufficiently high quality and resolution for industrial products have not yet been achieved, as the use of conventional printing techniques is severely limiting. Here we report a printing technique to manufacture ultrafine conductive patterns utilizing the exclusive chemisorption phenomenon of weakly encapsulated silver nanoparticles on a photoactivated surface. The process includes masked irradiation of vacuum ultraviolet light on an amorphous perfluorinated polymer layer to photoactivate the surface with pendant carboxylate groups, and subsequent coating of alkylamine-encapsulated silver nanocolloids, which causes amine–carboxylate conversion to trigger the spontaneous formation of a self-fused solid silver layer. The technique can produce silver patterns of submicron fineness adhered strongly to substrates, thus enabling manufacture of flexible transparent conductive sheets. This printing technique could replace conventional vacuum- and photolithography-based device processing.

## Abstract (web summary)

Silver nanocolloids are promising materials for printed electronic technologies. Here, the authors manufacture ultrafine conductive patterns utilizing the exclusive chemisorption of weakly encapsulated silver nanocolloids on a photoactivated surface.

## Graphical abstract

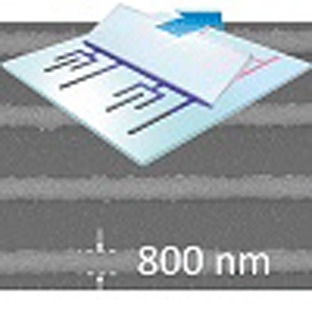

Metal nanoparticles can be densely suspended in dispersion media if the metals are encapsulated by insulating ligand layers^1^. These protect the active or unstable bare metal surfaces and preserve the high surface-area-to-volume ratio of the nanoparticles, preventing the self-aggregation^2^,^3^, coalescence^4^, welding^5^, decreased melting temperature^6^,^7^ or liquid droplet-like deformation, especially for silver nanoparticles (AgNPs) at room temperature^8^,^9^.

Concentrated silver nanocolloids, or nanometal inks, composed of encapsulated AgNPs have recently attracted considerable attention for applications in environmentally friendly printing-based device production (that is, printed electronics) technologies^10^,^11^,^12^,^13^,^14^,^15^. Printed electronics technologies are crucial in realizing a low-consumption society, as they save resources, energy and time in the creation of flexible, large-area and ambient devices. The large-scale production of monodispersed AgNPs has progressed considerably^16^,^17^,^18^,^19^. Several cases of direct chemical synthesis of encapsulated AgNPs were reported, utilizing thermolysis processing of precursor silver complexes, the ligands of which later served as encapsulating layers^18^,^19^. These techniques are promising for producing inks for printed electronics, as they may allow predesigned encapsulating layers with tunable coordination strengths while maintaining the equilibrium metastability of the ink.

Much effort has been dedicated to manufacturing fine metal wiring patterns by various printing methods, such as screen or inkjet printing, with nanometal inks^10^,^11^,^12^,^13^,^14^,^15^. However, the obtained printed patterns have not yet offered the material quality, pattern resolution, substrate compatibility or substrate adhesion required for most industrially available electronic devices^20^,^21^. These limitations result from the physisorption phenomena of the fluidic nanometal inks utilized. The inherent liquid nature of the inks results in unsatisfying resolution or quality of the printed deposits, such as non-uniform layer thickness due to the coffee-ring phenomenon^22^,^23^,^24^. The incompatibility of the encapsulating layer before and after the printing deposition is also critical. While the layer is necessary for stabilizing the nanocolloid, it must be removed after deposition to restore the metal conductivity. To remove the encapsulated layer, annealing is used to fuse the AgNPs at high temperatures, although this frequently creates fatal voids and cracks in the conductive circuits and distorts heat-sensitive flexible plastic substrates. Considering these difficulties and dilemma, an ideal method would restore the self-aggregating ability of bare AgNPs at predefined positions by selectively removing the ligand encapsulation. Such a method has not yet been reported.

In this study, we report an innovative printing principle to manufacture ultrafine conductive patterns through an exclusive chemisorption (that is, surface chemical reaction) of AgNPs that triggers the self-aggregating ability of AgNPs on predefined photoactivated areas of a solid surface. For this purpose, we utilize a stable, concentrated and low-viscosity silver nanocolloid composed of AgNPs encapsulated in alkylamine layers^19^. Alkylamine coordination is weaker than carboxylate coordination, as demonstrated by the sintering temperature of less than 150 °C. We use a photoactivated polymer surface with pendant carboxylate groups, which combine more strongly with bare silver surfaces. When the alkylamine-encapsulated fluidic silver nanometal ink contacts the photoactivated surface, self-fused solid silver layers form spontaneously and exclusively on the photoactivated surface under ambient conditions. This unique AgNP-based phenomenon enables the extremely simple printing of large-area electronic circuits with submicron resolution adhered strongly to flexible plastic substrates.

## Results

### Printing process and printed products

**Figure 1:**
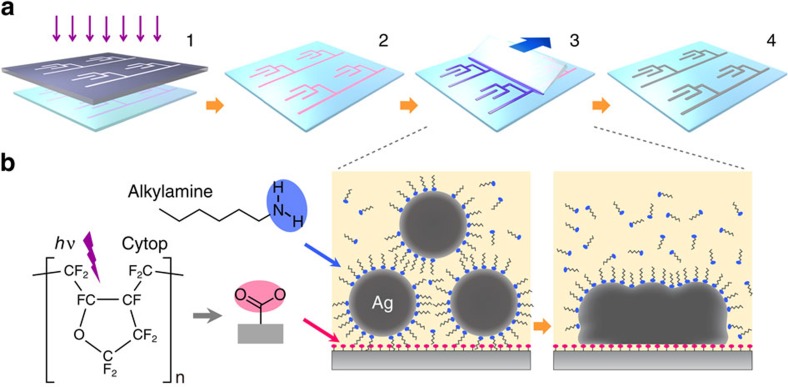
Procedure and principle of the printing method. (**a**) Schematic of the process. (**b**) Underlying mechanism.

**Figure 2:**
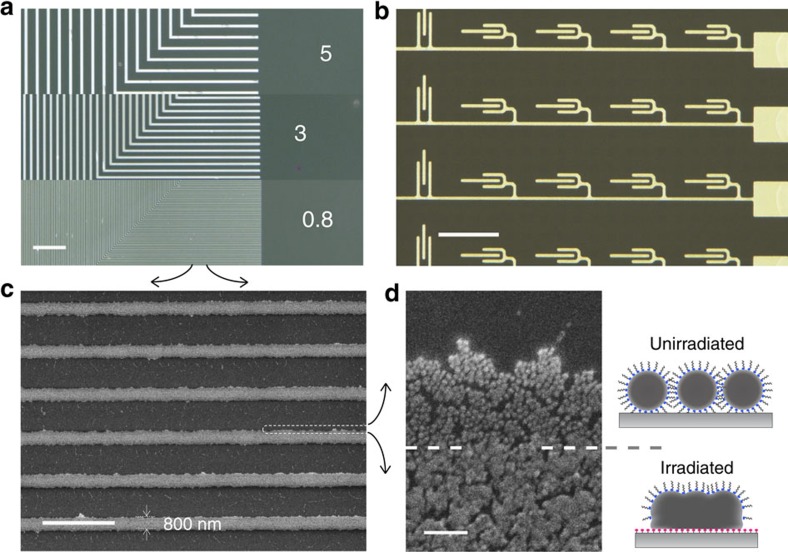
Products of the printing method. (**a**) Optical micrographs of printed patterns of parallel lines with widths of 5, 3 and 0.8 μm, respectively. (**b**) Optical micrograph of the printed silver pattern for source and drain electrodes of thin-film transistor arrays. (**c**) SEM image of the printed parallel lines with a width of 0.8 μm. (**d**) Expanded SEM image of the perimeter of the printed silver line pattern. Right: Schematic of fused and non-fused AgNPs. Scale bar: (**a**) 50 μm; (**b**) 100 μm; (**c**) 5 μm; and (**d**) 100 nm.

A schematic of the process is presented in Fig. 1a,b. To produce a patterned photoactivated surface, we used masked vacuum ultraviolet (VUV) irradiation on the amorphous perfluorinated polymer poly[perfluoro(4-vinyloxy-1-butene)] (Cytop)^25^,^26^. We fabricated thin films of Cytop by spin-coating it on silica, polyethylene naphthalate (PEN) or polyethylene terephthalate (PET) substrates. The spin-coated substrates were irradiated with VUV light at a wavelength of 172 nm through a photomask. The polymer surface was then exposed to the alkylamine-encapsulated silver nanometal ink (40–60 wt% dispersed in a 4:1 mixed solvent of *n*-octane and *n*-butanol) by blade coating under ambient conditions. A thin solid silver layer eventually formed on the irradiated parts of the polymer surface after the coating blade was swept, but the unirradiated parts remained bare (Supplementary Movie 1). As a result, fine silver patterns as shown in Fig. 2a–c were obtained. The highest resolution obtained is a line width of 800 nm. The resolution is 10-100 times higher than that obtained by conventional screen or inkjet printing^3^,^4^,^10^,^11^,^12^,^13^,^14^,^15^,^20^,^21^,^23^,^24^.

**Figure 3:**
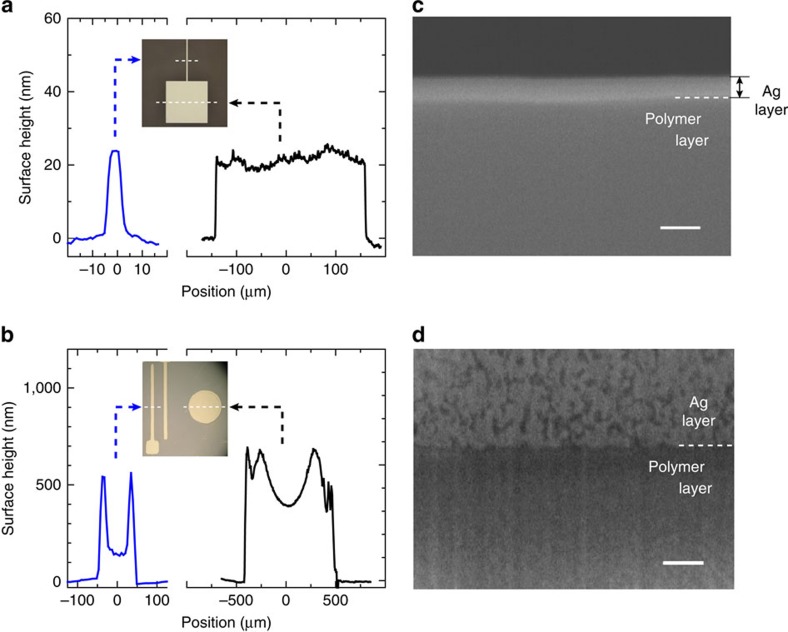
Comparison with conventional wet/dewet patterning. (**a**) Thickness profiles of the printed silver lines at different line widths by the present printing technique with silver nanometal ink with alkylamine encapsulation on photoactivated polymer surface, and (**b**) by conventional wet/dewet patterning with water-based silver nanometal ink with alkyl-carboxylate encapsulation. The profile in (**b**) presents typical thickness distribution due to the coffee-ring effect where the edge becomes thicker than the central region, while the profile in (**a**) presents flat distribution against the line cross section with no area-size dependence. (**c**) Enlarged SEM images of cross-section of the printed silver layer with 60 wt% alkylamine-encapsulated silver nanometal ink on photoactivated polymer surface, and (**d**) on unirradiated polymer surface. (**c**,**d**) Scale bar, 100** **nm.

The as-printed silver layer shows a high conductivity of ∼1.0 × 10^4^ S cm^−1^ with no post-treatment by heat. The conductivity gradually improves with time or by subsequent low-temperature annealing (<80 °C) to values reaching 1.0 × 10^5^ S cm^−1^. The thickness profile of the silver layer is flat against the line cross section, as presented in Fig. 3a. The silver layer is adhered to the substrate surface with an adhesive force exceeding 5 MPa. These characteristics differ from those of the silver layers obtained by conventional printing technologies. For comparison, we created a deposit on the unirradiated polymer surface by a contact casting technique. The dried deposit was easily peeled off the polymer surface. The surface contact angle of the dispersion solvent was ∼0° on the irradiated surface and 34.3° on the unirradiated surface (Supplementary Fig. 1). The difference in contact angle is similar to wet/dewet patterning using surface-energy-patterned substrates^27^,^28^. The relatively high dewetting nature is indispensable in achieving fine patterning, because the nanometal ink is briefly spread on but can be completely swept away from the unirradiated part during the process. Nonetheless, the technique is essentially different from typical wet/dewet patterning regarding the area-size dependence of thickness distribution (Fig. 3b) and the adhesive strength to the substrate surface.

**Figure 4:**
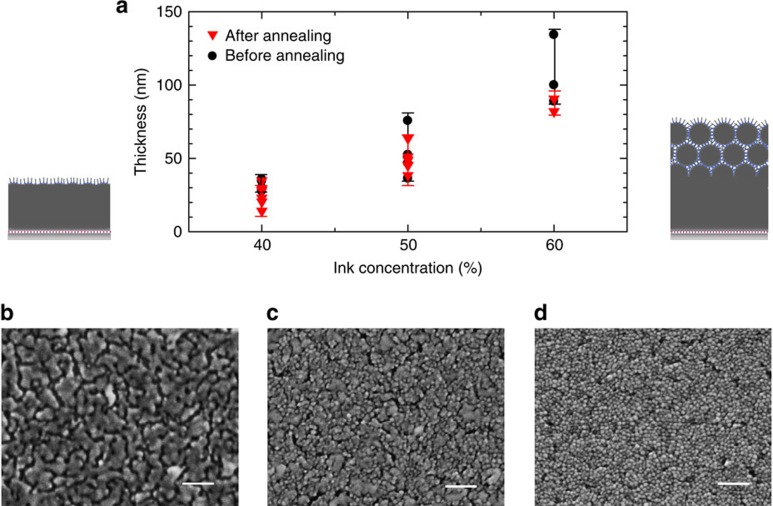
Ink concentration dependence of thickness and surface profiles of the printed silver layers. (**a**) Ink concentration dependence of thickness of the printed silver layers. Error bars represent the range of data. (**b**) Top views of the printed silver layers with different layer thicknesses obtained by printing nanometal ink with 40 wt%, (**c**) 50 wt% and (**d**) 60 wt% concentration, respectively. (**b**–**d**) Scale bars, 100 nm.

Figure 3c presents an enlarged SEM image of the cross-section profile of the printed silver layer (with 60 wt% nanometal ink) adhered to the irradiated polymer surface. The fused silver layer with a smooth interface with the polymer layer is formed, and the spherical profiles of the AgNPs have completely disappeared. The image indicates that the AgNPs are fully fused together, despite the low-temperature (<80 °C) processing. In striking contrast, the deposit produced by simple casting on the unirradiated polymer surface exhibits particulate features and voids near the interface with the polymer layer (Fig. 3d). The thickness depends not on the area size, but on the ink concentration (Fig. 4a). In the surface profiles of the printed silver layer (Fig. 4b–d), the presence of particulate features depends on the layer thickness: The surface SEM image of the thin silver layer printed with 40 wt% nanometal ink has no particulate features because complete self-fusion occurs, whereas particulate features dominate the surface of the thicker silver layer printed with 60 wt% nanometal ink. Note that the granular morphology observed in Fig. 4b is also observed in a thin silver layer fabricated by vacuum deposition on the same photoactivated Cytop film surface (see Supplementary Fig. 2). Furthermore, a clear transition from fusion to non-fusion is observed at the perimeter of the printed silver pattern (see Fig. 2b): The fused silver layer grown on the irradiated polymer surface is accompanied by a peripheral non-fused layer of spherical AgNPs. This indicates that a small amount of nanometal ink remains and dries on the neighbouring unirradiated area by capillarity, which creates the residue of the peripheral non-fused layer.

### Underlying mechanism of the printing process

**Figure 5:**
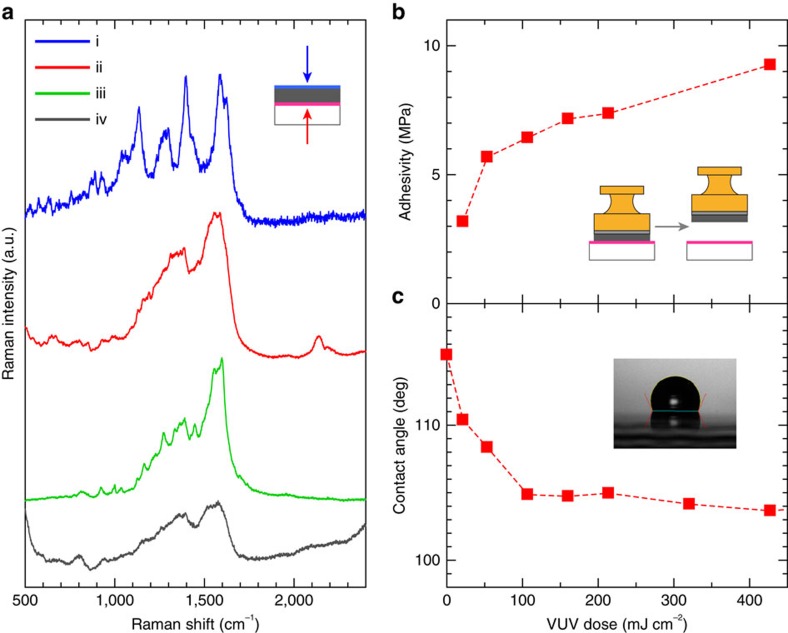
Evidence for chemical connection of the printed silver layer with photoactivated polymer surface. (**a**) Raman spectra (i) from the top surface and (ii) from the bottom interface of the printed silver layer on photoactivated polymer surface, (iii) from the bottom interface of the vacuum-evaporated silver layer on the photoactivated polymer surface, (iv) from the top surface of the deposit obtained by simple casting of the alkyl-carboxylate-encapsulated silver nanometal ink. (**b**) Adhesive strength of the printed silver layer to the substrate surface, and (**c**) water contact angle on the photoactivated polymer surface, both plotted as a function of the VUV irradiation dose.

To focus on the microscopic aspects of the silver-layer formation, Raman spectra as measured from the top surface of the silver layer and from the bottom interface with the irradiated polymer film through the transparent substrate are shown in Fig. 5a. Both sets of spectra exhibit prominent and distinctly different vibrational features. The measurement corresponds to surface-enhanced Raman spectroscopy, in which the ligand molecules coordinated (or adsorbed) to the AgNP surface can be sensitively detected^29^. The spectrum from the top surface exhibits four peaks at 1,135, 1,280, 1,395 and 1,590 cm^−1^ (Fig. 5a). These peaks likely relate to the alkylamine group, associated with NH_2_ deformation, CH_2_ deformation and wagging, and CN stretching vibrations; the observed features are much clearer than those of the former report^30^,^31^. Well-ordered alkylamine coordination to the surface of silver nano-structures may be responsible for the chemical enhancement of the surface-enhanced Raman spectroscopy signal. In contrast, two peaks at 1,360 and 1,570 cm^−1^ are seen from the bottom interface, which clearly differ from those from the top surface (ii). Similar features were reported for a carboxylate-coordinated silver surface^32^,^33^; the peaks were assigned to symmetric and antisymmetric COO^−^ stretching and CH_2_ wagging vibrations. Similar features appear in other cases, as shown in Fig. 5a; one is from the bottom interface of a silver layer formed by the vacuum evaporation of silver on the irradiated polymer surface (iii), while the other is from the top surface of the deposit obtained by casting of a different silver nanometal ink with alkylcarboxylate encapsulation (iv). The latter signal can be ascribed to the coordination of the carboxylate group to the AgNP surface. The observed two broad peaks are quite similar to those of (ii) and (iii). We conclude that the photoactivated area has pendant carboxylate groups, and that the bare surface of the evaporated silver film is likely coordinated by these carboxylate groups. Carboxylate groups on the photoactivated area should connect easily to bare AgNP surfaces at room temperature, because the encapsulating alkylamine molecules can be attached and detached at equilibrium conditions. We conclude that the alkylamine coordination is chemically converted to carboxylate coordination on the irradiated polymer surface.

**Figure 6:**
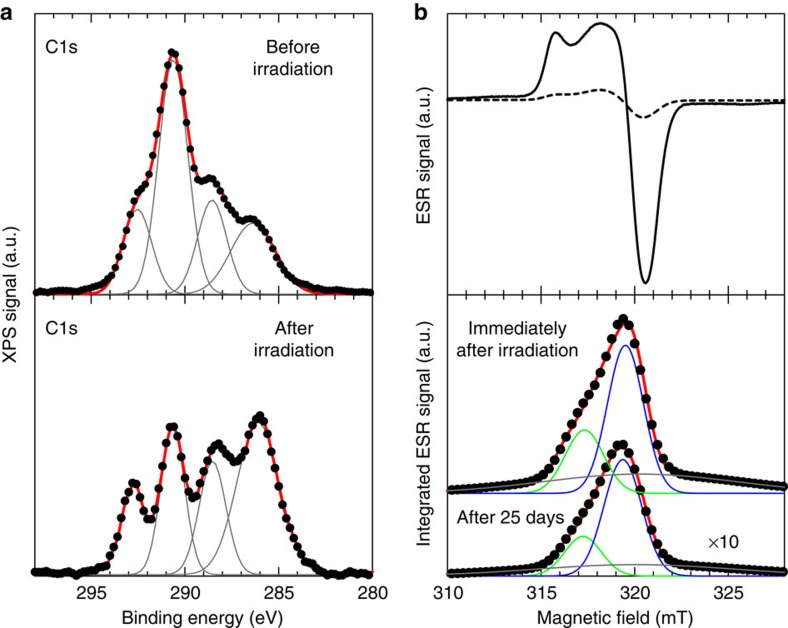
Spectral analyses of photoactivated polymer surface. (**a**) The carbon 1s XPS spectra are shown by black dotted curves for the spin-coated Cytop films before (upper part of panel) and after (lower part of panel) the VUV irradiation. It is clearly observed that the irradiation affords a decrease in the peak at 290.5 eV and an increase in the peak at 286.3 eV. The observed spectra were well fitted by the sum (red solid curve) of four Gaussian lines (gray solid curves for each). Among them, the largest peak at 290.5 eV can be assigned as difluoride carbons, while the peak at 286.3 eV can be assigned as the carbons in structures resulting from defluorination of the polymer. (**b**) First-derivative ESR spectra of a free-standing Cytop film just after the VUV irradiation with a dose of 512 mJ cm^−2^ (solid curve) and 25 days after the VUV irradiation (dashed curve). The irradiation affords appearance of the ESR signal due to peroxy radicals that are most probably formed by the reaction of carbon radicals with oxygen. ESR absorption spectra obtained by integrating the first-derivative signal are shown at the bottom. The upper panel is the spectrum of the film just after the VUV irradiation, while the lower panel is the spectrum of the film 25 days after the VUV irradiation. These spectra are well fitted by the sum (red solid curve) of three resonance components, respectively; the former with *g*-values at 2.0252 (green curve), 2.0113 (blue curve) and 2.0062 (grey curve), and the latter with *g*-values at 2.0249 (green curve), 2.0114 (blue curve) and 2.0071 (grey curve).

Regarding the origin of carboxylate group, VUV irradiation on perfluorinated polymers has been reported to cause photodegradation into several products, including carboxylic acid pendants on the polymer chain^34^,^35^,^36^,^37^. We obtained both X-ray photoelectron spectroscopy (XPS) and electron spin resonance (ESR) spectra of the Cytop films before and after VUV irradiation, as presented in Fig. 6. In the XPS spectra, the irradiation decreases the intensity of the peak at 290.5 eV and increases that of the peak at 286 eV (Fig. 6a). This spectral variation is ascribed to difluoride carbons (−CF_2_−) being converted to different de-fluorinated chemical species of carbon. The ESR spectra also demonstrate the generation of peroxy radicals (Fig. 6b). One possible photodegradation process is the cleavage of the ether bond (C−O) within the ring unit of the polymer to produce carbon radicals (−C) and acid fluoride (−CF=O) end groups by VUV irradiation. Through exposure to air, the former would be converted into peroxy radicals, while the latter would be followed by the hydrolytic formation of carboxylate groups. Thus, free carboxylate groups attached to the polymer chain are generated by VUV photodegradation on the surface of Cytop layers.

The irradiation-dose dependencies of the water contact angle on the irradiated polymer surface and of the adhesive strength of the printed silver layer to the substrate are shown in Fig. 5b. The decreased water contact angle by increasing the irradiation dose indicates the increased surface density of carboxylate groups. With increased irradiation, the adhesive strength of the silver layer to the substrate surface is increased considerably. High conductivity is obtained by the high-enough irradiation dose (∼100 mJ cm^−2^), while the conductivity becomes extremely low at low irradiation dose (∼20 mJ cm^−2^). We found that at this dose level the coated nanometal ink forms a network-like pattern on the surface (see Supplementary Fig. 3). We conclude that the printed silver layer is chemically bound to the polymer layer through carboxylate coordination.

**Figure 7:**
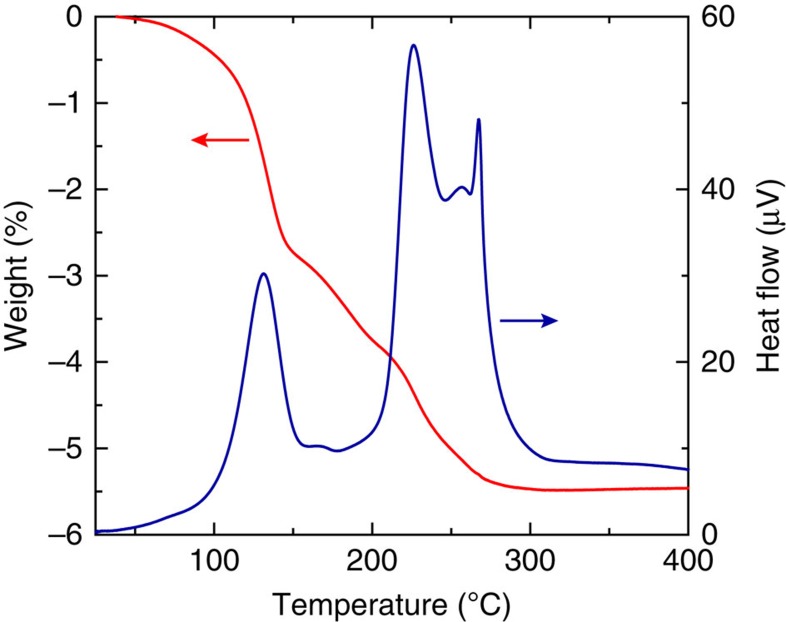
Thermal analysis of AgNP powder. TG-DTA curves for the dried alkylamine-encapsulated AgNP powders. We used the AgNP powder for the measurement immediately after the as-synthesized material was dried out under ambient conditions.

Based on all the observations above, we propose that when the alkylamine-encapsulated fluidic silver nanometal ink contacts the irradiated polymer surface with pendant carboxylate groups, it promotes the amine–carboxylate conversion for encapsulated AgNPs that triggers the spontaneous formation of a self-fused solid silver layer. The following mechanism may be responsible for the self-fusion process: as the alkylamine coordination is relatively weak, the alkylamine would repeatedly attach to and detach from the metal surface in the nanocolloid at room temperature. When the nanometal ink makes contact with the carboxylate-functionalized photoactivated surface, thermally moving AgNPs are captured by the carboxylate coordination when they collide with the photoactivated surface. When the trapped AgNP density becomes sufficiently high at the surface, the bare surfaces of the AgNPs make contact and fuse to form a solid silver layer. Actually, in the thermal analysis of dried alkylamine-encapsulated AgNP powder shown in Fig. 7, a notable exothermic reaction, accompanied by a simultaneous weight loss, was observed at a temperature lower than 100 °C and peaked around 130 °C. It is most probable that the weight loss should be due to the (endothermic) elimination reaction of alkylamine from the AgNPs, while it promotes the considerable exothermic fusion reaction between AgNPs. The exothermic nature of the fusion reaction would promote the cascade formation of a self-fused solid silver layer at room temperature. In particular, the exothermic process should raise the local temperature at the top Ag surface, which would make the alkylamine group detached more from the surface and thus promote additional adsorption of AgNPs on the roughly bare Ag surface. We consider that the scenario is quite consistent with the ink concentration dependence of the layer thickness, as presented in Fig. 4a; presence of many more AgNPs in the vicinity of the Ag surface should promote the adsorption of more AgNPs. Additionally, the mobile nature of ligand molecules in the fluidic ink would effectively eliminate the ligand molecules from the self-fused silver layer, which differs from the usual sintering process for aggregated AgNPs.

The ink stability can be kept good enough, in spite of the relatively weak alkylamine encapsulation and concomitant facile chemisorption on the photoactivated surface. We consider that the following facts should be a clue for understanding the peculiar nature of the alkylamine-encapsulated silver nanometal ink: Ink stability can be acquired at sufficiently high concentration (for example, at 50 wt%). However, the stable dispersion is lost if it is diluted; AgNPs easily aggregate in the diluted ink at 1.25 wt% (see Supplementary Fig. 4). It is most probable that the high alkylamine concentration in the high-concentration ink is indispensable for achieving equilibrium colloidal states of the nanometal ink where the alkylamine can repeatedly attach to and detach from the Ag surface in the ink.

### Printing flexible and transparent conducting electrodes

**Figure 8:**
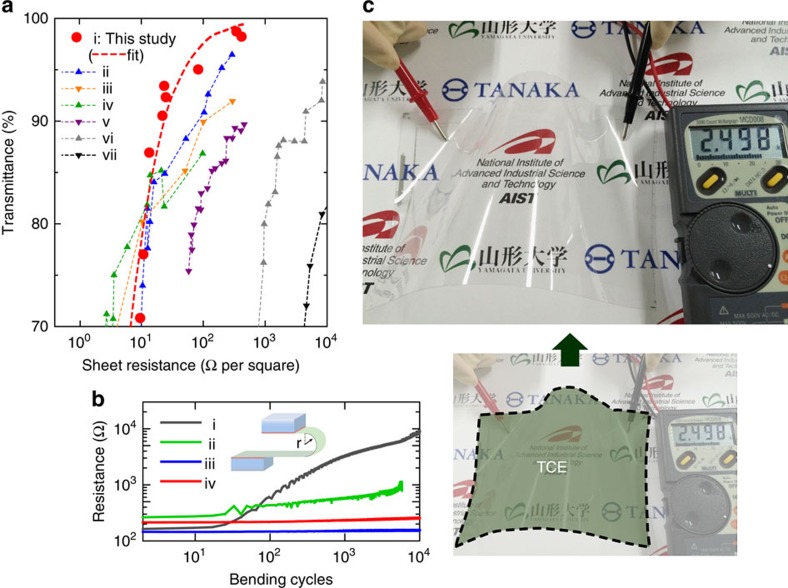
Application of the printing technique to touch-screen sensor technology. (**a**) Plot of transmittance vs. sheet resistance for the printed silver meshed line pattern with different line widths. Plots for other transparent conductor films (ii: copper nanowires (CuNWs), iii: indium tin oxide (ITO), iv: silver nanowires (AgNWs), v- PEDOT:PSS, vi: single-walled carbon nanotubes (SWNTs), vii: graphene) are shown for comparison^39^,^40^. (**b**) Results of bending test for the printed silver meshed line pattern; resistivity is plotted as a function of the number of bending cycles at various concave (i) and (iii) and convex (ii) and (iv) bending radii (2.5 mm in i and ii, and 5.0 mm in iii and iv). (**c**) Photograph of an 18-cm-wide capacitive-type transparent flexible touch-screen sensor sheet, fabricated with a PET substrate.

The present technique enables the print production of submicron-resolution conductive patterns through the reaction of the nanometal ink with the photoactivated polymer surface. We call the process surface photoreactive nanometal printing. The technique is applicable to the fabrication of many electronic devices. Transparent conducting electrodes (TCEs) used for touch screen technologies are one such class of devices, since TCEs can be manufactured with only narrow conductive metal wires if the line width of the wire is comparable to the diffraction limit of visible light. To investigate the performance of printed patterns as TCEs, two critical performance criteria of sheet resistance (*R*_s_) and transmittance (*T*) are plotted in Fig. 8a, in comparison to other representative TCEs^38^,^39^,^40^. As shown, the present technique provides the highest-performing TCEs, including the most widely used indium tin oxide TCEs. The obtained TCEs also present a higher tolerance to bending than the PEN substrate alone, as presented in Fig. 8b: The increased resistance in plots with 2.5 mm bending radius at >10^2^ bending cycles is caused not by the deterioration of the printed silver pattern, but by the degradation of the PEN substrate. It clearly demonstrates the applicability of the technique for flexible electronics. We manufactured a capacitive-type touch-screen sensor of width ∼18 cm (with a touch sensor line width of 2 μm at intervals of 300 μm, and frame line width of 50 μm) on a PET sheet, as depicted in Fig. 8c. Sensing operations were successfully performed with the sensor sheet (Supplementary Movie 2).

## Discussion

The printing technique is simple, consisting only of masked VUV irradiation and blade coating of alkylamine-encapsulated nanometal ink. The method utilizes a ligand conversion (chemisorption) for weakly encapsulated AgNPs that triggers the self-fused formation of high-resolution patterned silver layers adhered strongly to the substrate. The achieved resolution and quality has previously been impossible to produce by printing techniques based on physisorption phenomenon of the fluidic inks. Considering the VUV wavelength, the resolution could be improved further. The process does not require a vacuum atmosphere, which is a main source of energy consumption in the production of electronic devices. Additionally, the consumption of nanometal ink can be minimized (to as small as ∼1–3 μl for a 10-cm^2^ substrate), as almost all silver contained in the ink is used to transform into metal wires. The method is particularly promising as a next-generation technology for producing electronic products with flexibility, large areas and arbitrary shapes, as well as for the exploration of applications in which thin films or wiring patterns of precious metals are needed.

## Methods

### Preparation of silver nanometal ink

AgNPs were synthesized by thermal decomposition of silver oxalate, Ag_2_(C_2_O_4_), as reported in the literature^19^. The silver oxalate was activated by alkylamines (R-NH_2_) via the formation of oxalate-bridged silver alkylamine complexes, [(R-NH_2_)Ag(*μ*-C_2_O_4_)Ag(R-NH_2_)]. The oxalate-bridged silver alkylamine complexes underwent low-temperature decomposition at 110 °C with evolution of CO_2_, and alkylamine-encapsulated AgNPs were generated in an almost quantitative yield. Various alkylamines were adopted to activate the silver oxalate in order to prepare a stable, concentrated and low-viscosity silver nanometal ink. In a typical case, *N*,*N*-dimethyldiaminopropane, hexylamine and dodecylamine were mixed along with a trace amount of oleic acid to synthesize AgNPs that are dispersed independently in a mixed solvent of *n*-octane and *n*-butanol (4:1 in volume). The AgNPs are in spherical shape, with a mean dimension of 13.6 nm and a narrow size distribution (*σ*=1.1 nm). In this study, silver nanometal inks were prepared in the concentration range between 40 and 60 wt%.

We also used silver nanometal ink with alkylcarboxylate encapsulation for reference measurements (Fig. 3b). The ink was obtained by thermal decomposition of silver dodecanoate (AgC_12_H_24_COO), according to the literature^41^. The alkylcarboxylate-encapsulated AgNPs are spherical in shape, with a mean dimension of ∼5 nm.

### Fabrication of perfluorinated polymer layer

We used a silica plate, PEN or PET film as the base substrate. A thin layer of amorphous perfluorinated polymer, poly[perfluoro(4-vinyloxy-1-butene)] (Cytop), was fabricated on the substrate by spin coating of diluted solution in fluorinated solvent (CTL-809M, Asahi Glass Co., Ltd., Japan) at 2,000 r.p.m. for 60 s at room temperature. Then the films on silica were dried at 180 °C for 60 min, and those on PEN or PET films were dried at 80 °C in vacuum for 60 min.

### Masked irradiation of VUV light

We used a UV dry processor (VUS-3150, ORC Manufacturing Co., Ltd. Japan) for irradiating VUV light by an Xe_2_ excimer lamp. The films were set in a chamber filled with N_2_ gas with residual O_2_ lower than 300 p.p.m. Uniform irradiation is achieved by periodic motion of the sample stage with a period of 20 s. The average dose rate was estimated at 6.4 mW cm^−2^, and the irradiated power density was adjusted with a filter (synthesized silica plate coated with thin Cr) and irradiation duration. A patterned photoactivated polymer surface was produced by masked VUV irradiation through a photomask composed of a predefined mask pattern of Cr on a synthesized silica plate.

### Characterization of photoactivated polymer surface

Contact angle measurements were conducted with a contact angle and surface tension analyser (FTA188; First Ten Angstroms, Inc., USA). Atomic force microscope images were obtained with a scanning probe microscope (Dimension 3000 Nanoscope IIIa; Bruker Co., Ltd., USA). XPS measurements were carried out using an XPS apparatus (ULVAC-PHI 5000, ULVAC Inc., Japan) with monochromatic Al K_α_ radiation (1,486.6 eV, 15 kV). Because the Cytop layer is highly insulating and easily charged up by an incident beam, the films were slightly coated by vacuum-evaporated Ag to cover the partially polymer surface by island-shaped Ag. All the measurements were conducted with a neutralizer, and the offset shift was corrected by the reference signal of Ag 3d_5/2_ (=368.3 eV). ESR measurements were carried out using an X-band ESR apparatus (JES-FA200, JEOL Ltd., Japan). A thick Cytop film with a thickness of ∼25 μm was fabricated by repeated (eight times) spin coating on a silicon wafer, and was peeled off from the wafer. The free-standing film was used in the ESR measurement. For this purpose, we used another Cytop (CTX-809SP2, Asahi Glass Co., Ltd.), which enables the facile peeling of Cytop film from the wafer. We could not detect any ESR signal from the unirradiated polymer film.

### Coating of silver nanometal ink

We used a bar coater (AB3125, The Paul N. Gardner Company, Inc., USA) for the blade coating of silver nanometal ink on substrates with photoactivated polymer surfaces. We first fixed the substrate on a table, and deposited two or three drops of nanometal ink on the substrate surface near its edge. Then the line edge of the blade composed of smoothly polished glass-plate edge was put gently on the drops, and the blade was swept over the substrate surface at typical sweep rate of 2 mm s^−1^.

### Characterization of the printed silver layer

We used a digital microscope (VHX-5000; Keyence Co. Ltd., Japan) for the optical microscope observations. The SEM images were obtained with a scanning electron microscope with a field-emission gun (JSM-7000 F or -7500 F; JEOL Ltd.). The samples for cross-sectional SEM observation were prepared by an ion slicer (IB-09060CIS; JEOL Ltd.), keeping the sample temperature at −150 °C. Electrical properties of the printed silver patterns were measured by four-terminal measurements using a semiconductor parametric analyser (E5270A; Agilent Technologies Co. Ltd., USA). The thickness profile was estimated by using a stylus profiler (Alpha-Step D-500; KLA-Tencor Co. Ltd., USA) or by a scanning probe microscope (Dimension 3000 Nanoscope IIIa; Bruker Co., Ltd.). Raman measurements were carried out using a confocal Raman microscope (inVia, Renishaw Co. Ltd., UK). The adhesion measurements were carried out using a pull-off adhesion tester (PosiTest AT-A; DeFelsko Co., USA), conformable to ISO 4624.

### Characterization as a flexible TCE

We fabricated various silver wire grid patterns at uniform intervals of 250 μm with different line widths (15 kinds between 0.8 and 100 μm) by the present surface photoreactive nanometal printing technique on a silica substrate. Transmittance is estimated with a UV–vis spectrometer (U-3500; Hitachi High-Tech Co. Ltd., Japan) by averaging the transmittance in the wavelength range between 400 and 800 nm. Bending test, conformable to JIS C5016, is carried out using a flexing tester (FPC; Yasuda Seiki Seisakusho Ltd., Japan) for the printed silver layer on a PEN substrate (Q65HA, Teijin Dupont Films Ltd., Japan). The moving cycle was set at 0.72 s. The measurements were terminated due to the generation of cracks in PEN substrate itself at the bending radii of 2.5 mm.

## Additional information

**How to cite this article:** Yamada, T. *et al*. Nanoparticle chemisorption printing technique for conductive silver patterning with submicron resolution. *Nat. Commun.* 7:11402 doi: 10.1038/ncomms11402 (2016).

## Supplementary Material

Supplementary Information Supplementary Figures 1-4

Supplementary Movie 1 The movie shows how to expose silver nanometal ink by the blade coating technique on a flexible substrate with photoactivated patterned polymer surface. Few drops of the ink is put on the surface, and then the blade is swept at a constant velocity, which leads the conductive ultrafine silver pattern to emerge on the patterned photoactivated polymer surface. Optical microscope observations are also shown for the printed silver patterns with line and space (L/S) at 4μm/12μm, 3μm/9μm, 2μm/6μm, and 1μm/3μm.

Supplementary Movie 2 The movie shows an appearance, microscope observations, and an operation of a 8-inch-wide capacitive-type flexible touch screen sensor sheet produced by the SuPR-NaP technique.
