# Supplementary material for: Nanoparticle chemisorption printing technique for conductive silver patterning with submicron resolution
Source: Nat Commun. 2016 Apr 19;7:11402. doi: 10.1038/ncomms11402 (PMC4838887; doi:10.1038/ncomms11402)
Supplement: Supplementary Information — Supplementary Figures 1-4 [file ncomms11402-s1.pdf]

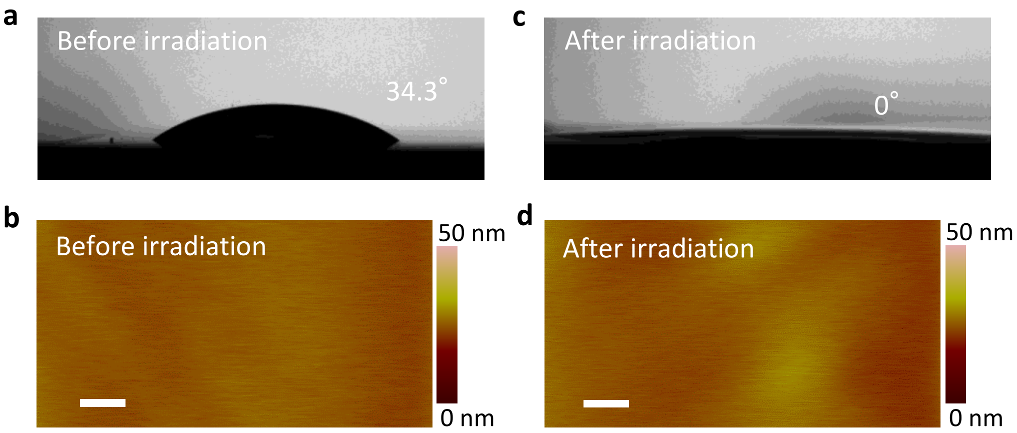

Supplementary Figure 1 | Wettability of photoactivated polymer surface. (**a**) Contact angle image of dispersion media (4:1 mixed solvent of *n*-octane and *n*-butanol) on the spin-coated Cytop® film surface before VUV irradiation, and (**b**) surface profile AFM image on top of the Cytop® film surface before VUV irradiation. (**c**) Contact angle image after VUV irradiation (wavelength at 172 nm and power density at 130 mJ cm^{-2}), and (**d**) surface profile AFM image after the VUV irradiation. The contact angle is 34.3° before the irradiation, and reduces to almost 0° by the irradiation, whereas no change is observed in the surface profiles at least at low irradiance level. (We observed an irradiation-induced hollow by the irradiance at higher than 1 J cm^{-2}.) The scale bars in **b** and **d** are 5 μm.

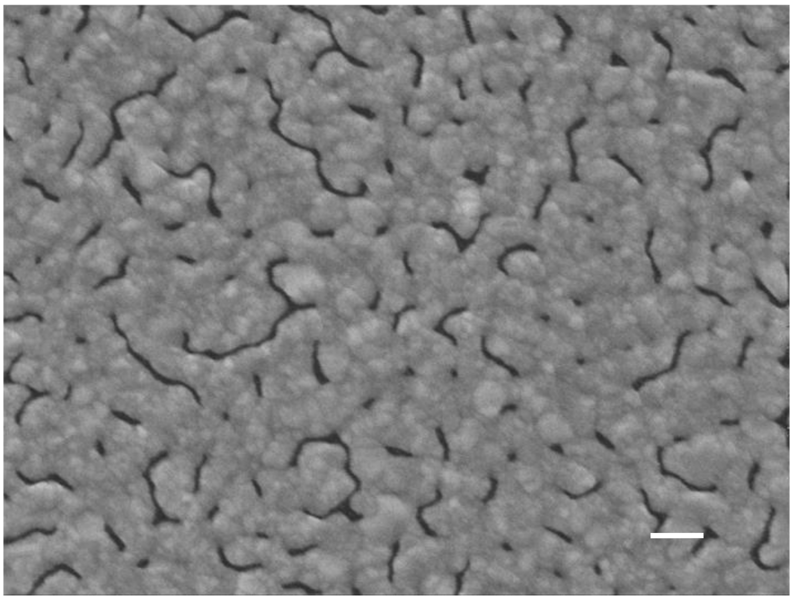

Supplementary Figure 2 | SEM image of evaporated silver film. The image shows SEM image of 30 nm-thick silver film fabricated by vacuum deposition on a photoactivated Cytop® film surface. We observed similar granular morphology with several vacancies in the film as in the 30-nm thick printed silver layer presented in Fig. 4b. The result indicates that the feature could be ascribed to the columnar growth of silver. The scale bar is 100 nm.

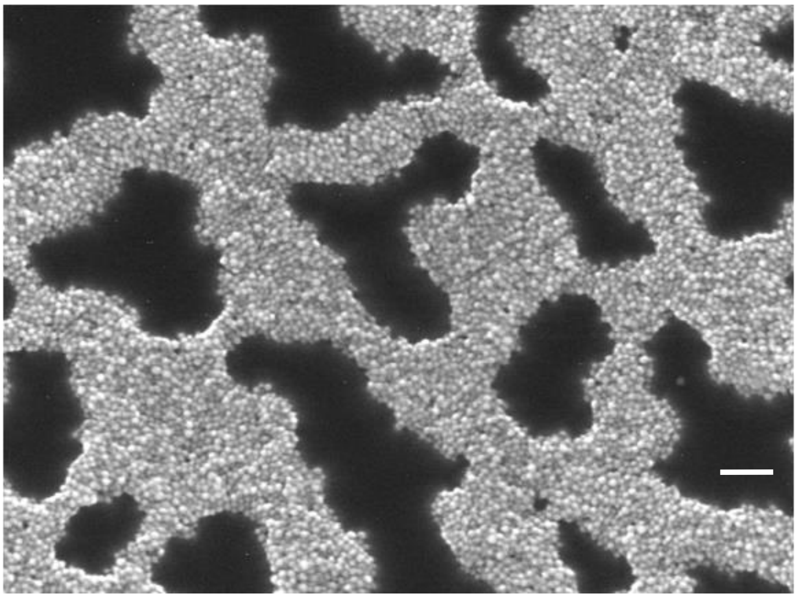

Supplementary Figure 3 | SEM image of printed silver layer on weakly-photoactivated surface. When the silver nanometal ink is coated on the weakly-photoactivated Cytop® film with much lower VUV irradiation dose at 20 mJ cm^{-2}, conductivity becomes extremely lower than that obtained with usual dose level (~100 mJ cm^{-2}). We found that at this dose level the coated nanometal ink forms network-like pattern on the surface as presented in this SEM image; the pattern morphology should be dominated by the aggregation of AgNPs rather than by the adsorption of AgNPs on the photoactivated surface. The scale bar is 100 nm.

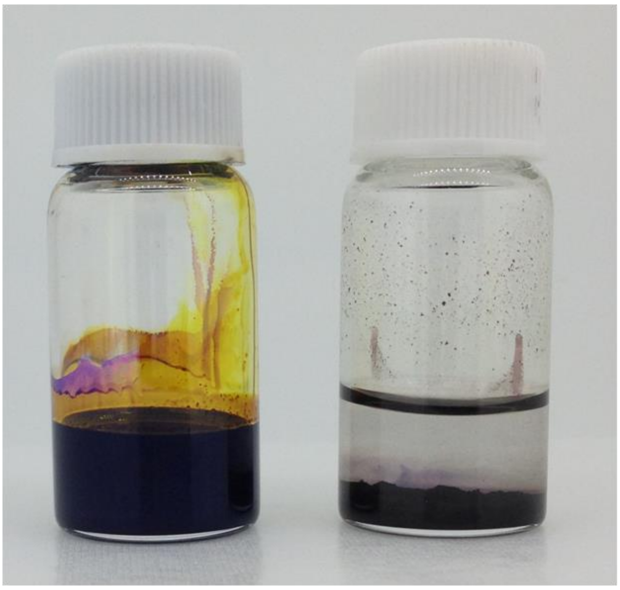

Supplementary Figure 4 | Appearance of alkylamine-encapsulated silver nanometal ink. Left: silver nanometal ink at 50 wt%. Right: at 1.25 wt%. This image indicates that the ink stability is acquired only at enough high concentration, while stable dispersion becomes difficult when it is diluted.
